# Exercise interveNtion outdoor proJect in the cOmmunitY—ENJOY program for independence in dementia: a feasibility pilot randomised controlled trial study protocol

**DOI:** 10.1186/s40814-022-01027-x

**Published:** 2022-03-22

**Authors:** Pazit Levinger, Anita M. Y. Goh, Jeremy Dunn, Josephine Katite, Ritu Paudel, Adrian Onofrio, Frances Batchelor, Maya G. Panisset, Keith D. Hill

**Affiliations:** 1 National Ageing Research Institute, Royal Melbourne Hospital, PO Box 2127, Melbourne, VIC 3050 Australia; 2 Institute for Health and Sport, Victoria University, Melbourne, Australia; 3 Rehabilitation, Ageing and Independent Living (RAIL) Research Centre, Monash University, Melbourne, Australia; 4 The University of Melbourne, Melbourne, Australia; 5 Old Colonists’ Association of Victoria, Melbourne, Australia

**Keywords:** Dementia, Cognitive decline, Physical activity, Falls, Built environment, Age-friendly, Seniors Exercise Park

## Abstract

**Background:**

While the underlying neuropathology of dementia is not curable, interventions and treatment, such as physical activity, can offer physical and functional gains leading to better mobility, independence and quality of life. The Seniors Exercise Park program is an evidence-based physical and social activity program using an innovative design in outdoor exercise equipment specifically designed for older people. This unique program has never been tested with older people living with dementia. This study will evaluate the feasibility of delivering the Seniors Exercise Park program for people living with mild to moderate dementia in residential aged-care. This study will identify the optimal physical activity program, evaluate the safety of equipment usage and determine optimal supervision needs. The potential physical, social, quality of life and cognitive benefits of participation in the Seniors Exercise Park program will also be examined.

**Methods:**

This is a feasibility pilot randomised controlled design with pre-post evaluation. Adults aged ≥ 60 years who have symptoms of dementia and/or who have been diagnosed with dementia will be recruited from an aged-care facility in Melbourne. Participants allocated to the intervention group will undergo a 12-week structured supervised physical activity program using the outdoor Seniors Exercise Park equipment followed by a 12-week maintenance phase (unstructured physical activity). Participants will be assessed at baseline, 3 and 6 months. Participants allocated to the control group will attend activities provided by the aged-care facility. A sample of 12 participants per group is the targeted sample size. Feasibility will be evaluated in terms of recruitment rate, retention, attendance, overall adherence, dropout rate, adverse events, modifications to the exercise program delivery and supervision needs. A comprehensive suite of cognitive and health-related questionnaires and physical function measures will also be collected.

**Discussion:**

The ENJOY program for independence in dementia will determine the suitability of the Seniors Exercise Park program for people diagnosed with mild to moderate dementia. Outcomes could inform future design of dementia-friendly built environments to increase physical activity participation for residential aged-care facilities.

**Trial registration:**

This trial is registered with the Australian New Zealand Clinical Trials Registry—Registry Number ACTRN12620000733976. Registered on the 13th of July 2020.

## Introduction

The world’s population is ageing rapidly, with the number of people aged over 65 years doubling to around 25% of the total population over the next 40 years. The number of Australians aged 65 and over is expected to increase from around 2.5 million in 2002 to 6.2 million in 2042 [1]. An estimated 459,000 Australians had dementia in 2020 [2], 99% of whom were aged 60 and over [3]. The total number of people affected by dementia is projected to rise to 900,000 by 2050. In Australia in 2015, almost half (49%) of people living in residential aged-care had a diagnosis of dementia and nearly all had an associated disability [3].

Participation in physical and social activities as part of quality dementia care is crucial for optimising the health and independence for people living in aged-care facilities. There are no disease-modifying treatments for dementia, so interventions that assist in the management of symptoms, such as physical activity, can offer physical and functional gains leading to better mobility, independence and quality of life. Physical activity offers many health benefits for older people, including improvement in cognitive function [4]. Less than 25% of older Australians do enough physical activity to achieve health benefit, and those living in residential aged-care spend the majority of their time inactive with lengthy periods of sedentary behavior [5, 6]. Furthermore, many have one or more functional dependencies and require assistance with daily living activities or ambulation. Exercise and physical activity programs that target daily life mobility-specific activities (e.g. sit to stand, transfer movements), have been shown to be effective in improving mobility limitations in older people living with dementia [7]. Novel enjoyable exercise programs that can motivate people living with dementia in aged-care facilities to engage regularly in physical activity are needed.

Being outdoors provides important benefits for older people [8, 9]. People living with dementia also showed benefits from being outdoor as it provides both physical and psychological health benefits as well as calming effect [10–12]. It may also improve cognition [13]. Encouraging older people to spend time outdoors might be effective in preventing cognitive decline among those with limited physical function, as going outdoors less than once a week was associated with cognitive decline among older adults with limited physical function [14]. Combining physical activity in outdoors environments might have added benefits for people living with dementia.

The Seniors Exercise Park is an innovative design in outdoor exercise equipment, integrating multiple exercise stations specifically designed for older people. This unique outdoor equipment offers a fun but physically challenging environment to support exercise and to challenge key aspects of physical health for older people, including balance (e.g. unstable walking bridge, narrow walking beam), mobility (e.g. shoulder range of movement), strength and function (e.g., sit to stand, stairs) [15]. In our previous randomised controlled trial, we tested the feasibility and effectiveness of using the Seniors Exercise Park as part of a physical activity and social support program (the Seniors Exercise Park program) for older people (without dementia or cognitive impairment). This research has provided the initial evidence demonstrating the physical and social benefits of the Seniors Exercise Park program for older people [16, 17]. Furthermore, in order to investigate its potential wider usage and health benefits in the community, a large community research project was undertaken 2018–2020 (the ENJOY project, 95 participants recruited). The ENJOY project involved the installation of the specialised outdoor equipment at public spaces and in an aged-care setting, together with the delivery of the evidence-based physical and social activity program for older people. The results from the ENJOY project further demonstrated the positive impact of the social and physical activity program on sustained engagement in physical activity and wellbeing and highlighted this novel approach as an important public health infrastructure investment in promoting physical activity for older people without cognitive impairment [18, 19].

Despite the positive health benefits reported, this innovative outdoor physical activity and social program has never been tested on and applied to people living with dementia. Therefore, the ENJOY program for independence in dementia aims to design, deliver and test this program in older people living with dementia in a residential aged-care. The optimal physical activity program, its safety and supervision needs will be examined to determine its suitability for people diagnosed with mild to moderate stage dementia. In addition, the physical, social, health and cognitive benefits of participation in the Seniors Exercise Park program will also be examined.

## Method and design

All procedures involved in this trial will be conducted in compliance with the National Statement on Ethical Human Research and the Australian Code for the Responsible Conduct of Research. Ethical approval has been obtained from the Melbourne Health Ethics Committee, Melbourne, Australia (HREC/61926/MH-2020). The study was designed according to the Consolidated Standard of Reporting Trials (CONSORT) guidelines and publications associated with the trial will be reported according the CONSORT 2010 Statement [20, 21].

### Design and setting

**Fig. 1:**
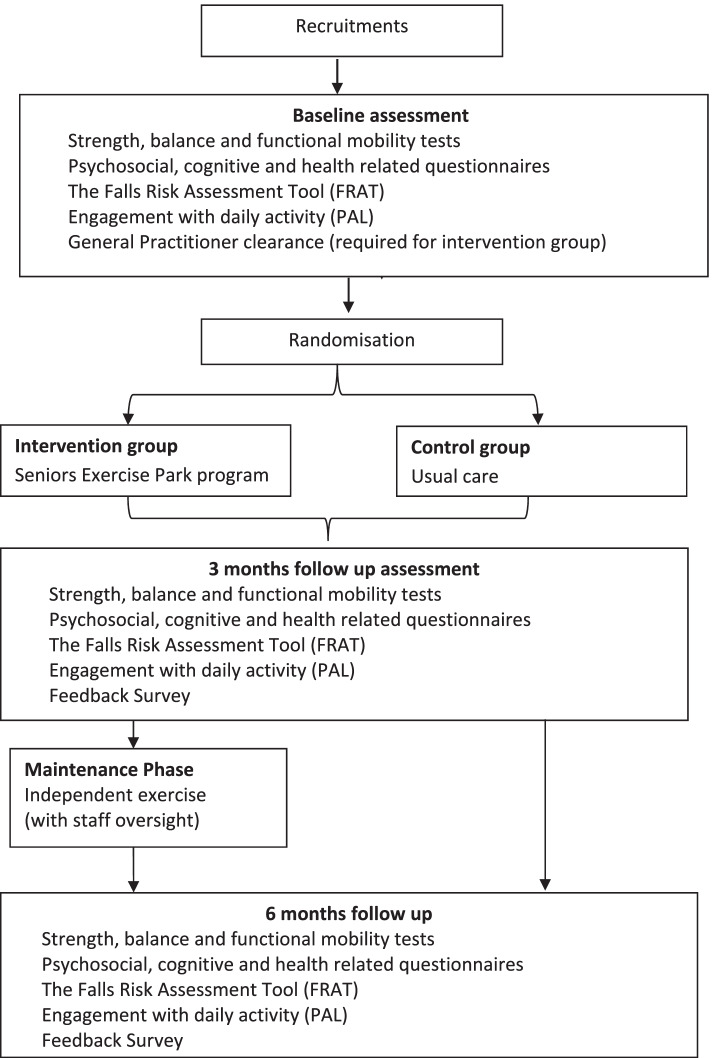
Chart flow of the ENJOY program for independence in dementia project’s design

This study is a feasibility pilot randomised controlled trial with pre-post evaluation. Older people aged 60 years and over who reside in Leith Park aged-care facility in Melbourne and who experience symptoms of dementia (such as memory loss or forgetfulness) consistent with a diagnosis of dementia and/or have a formal diagnosis of dementia will be recruited. Participants will be randomised to either an exercise intervention group (Seniors Exercise Park program) or to a control group. Participants from the intervention group will undergo a 12-week supervised structured physical activity program using the Seniors Exercise Park outdoor equipment, followed by a 12-week maintenance phase (unstructured independent exercise). Participants from the control group will be given the opportunity to participate in organised recreation and leisure-based group activities such as art and music (as part of the aged-care facility’s standard care). All participants will be assessed at baseline, 3 months and 6 months follow-up as detailed in Fig. 1.

### Study population

#### Inclusion criteria

Older people from the Leith Park aged-care facility will be recruited if they meet the following inclusion criteria: (1) aged 60 years or older and reside at the aged-care facility; (2) have been diagnosed with mild to moderate dementia, OR suspected to have dementia by the aged-care staff and a screening test score of Standardised Mini-Mental State Examination (sMMSE > 10), OR have cognitive problems that, in the opinion of the experienced staff, puts them in the cognitively impaired domain with suspected mild to moderate dementia, and a screening test score of sMMSE > 10 [22]; (3) are able to stand by themselves with or/without hand support; (4) are able to walk without manual (physical) assistance from staff and; (5) are able to follow simple exercise instructions.

#### Exclusion criteria

Older adults will be excluded from this study as follows: (1) participants who are unable to stand by themselves with or without hand support; (2) unable to walk without manual (physical) assistance from staff (with or without walking aid); (3) unable to comprehend simple instructions during the exercise program (this exclusion criterion will be determined by the intervention health professional within the first two classes, based on their observation of each participant in the first two sessions); (4) have severe dementia (score on the sMMSE ≤ 10) [22]; (5) score on the sMMSE > 24 (6) participants with other terminal or unstable illness or chronic conditions, or any documented medical condition or physical impairment that is deemed by their medical practitioner to contraindicate their inclusion.

### Recruitment and consent process

Information about the study will be provided to residents and their family members by the aged-care staff via verbal communication, flyers placed at reception and letters sent to family. Potential participants (who have been diagnosed with dementia or suspected to have dementia) will also be identified by the aged-care staff. This will be done based on their knowledge of the resident and the resident’s medical record file. Those who are diagnosed or suspected to have mild to moderate dementia (with a sMMSE score of > 10) will be given information about the study. Details of potentially eligible and interested participants will be provided to the research staff, who will then follow up with a phone call (to the family member/nominated representative) or in-person via a visit at the aged-care facility to further ascertain the resident’s interest to participate, and their capacity to consent. During that visit, the researchers will provide the resident with the information sheet. If it is deemed that the participant has the capacity to consent, an appointment will be made for the baseline testing. If it is determined that the resident does not have capacity to consent, but could participate in some or all parts of the project (able to answer questionnaires and follow exercise instructions), the research team will contact the nominated representative of the resident (as defined by the legislation in the relevant jurisdiction). The nominated representative will be asked to sign a consent form along with assent from the resident.

### Randomisation

Randomisation (1:1 basis) will take place after completion of the baseline testing. Participants will be randomly allocated to one of the following groups: (1) control group or (2) Seniors Exercise Park program group. Randomisation will be stratified by dementia severity (moderate or mild) based on the Standardised Mini-Mental State Examination (moderate sMMSE > 10 but < 20; mild sMMSE ≥ 20 but ≤ 24) [22]. Block randomisation will be undertaken using opaque envelopes, so that blocks of 6–8 participants (3–4 for intervention group and 3–4 for control group) will be randomised at a time. It is expected that recruitment will be undertaken over a period of 6–10 months to achieve a target sample size of 12 participants in each group. Assessors and participants will not be blinded to their respective group allocation. While there are clear benefits from a research perspective to blind assessors, having the same staff undertake assessments and interventions may reduce confusion and improve relationships for the person with dementia where a minimum number of research staff are involved.

### Procedure

At the baseline assessment, the following information will be recorded via semi-structured interview with the participant: demographic characteristics (age, gender), anthropometric measures (height and weight), previous medical history, current medication usage, socioeconomic and cultural background information (e.g. employment, level of education, country of birth, years of residency in Australia) and falls history (number of falls in the past 12 months). Information about medical history, medication usage, dementia diagnosis and falls history will also be extracted from the participant’s medical record kept onsite, to cross-check and to optimise accurate data collection.

At baseline, 3 months and 6 months participants will undergo a comprehensive suite of physical function (strength, balance, functional mobility) tests, psychosocial (quality of life, loneliness, depression) questionnaires, falls risk assessment and falls history assessments. Randomisation will take place at the completion of the baseline assessment. Those who are randomised to the exercise intervention group will be required to obtain a medical clearance from the General Practitioner on-site prior to commencing participation in the exercise program. Study design and study procedures and assessments are provided in Fig. 1 and Table 1 respectively.

**Table 1:**
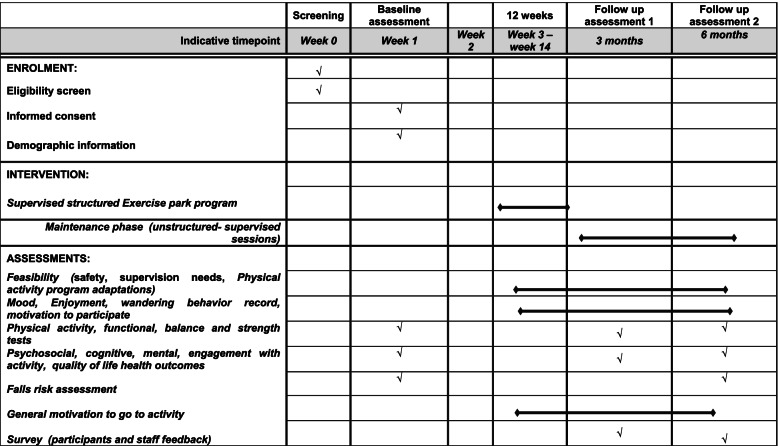
Study schedule

The assessments at baseline, 3 months and 6 months will take approximately 1–1.5 h and be conducted at a room at the Leith Park aged-care facility (Liscombe House). Participants will be given as many breaks as needed during the assessment and will also be able to complete it in two visits if requested and/or the resident reports fatigue.

### Assessments

#### Primary outcome

The primary outcomes for this study are the feasibility, safety and supervisory needs associated with the program:

##### Feasibility

Feasibility of the program will be determined based on the following: recruitment (% approached who agreed to participate), completion of the intervention (retention), attendance, overall adherence, dropout rate, adverse events (falls, muscle/joint pain), any modification in the exercise program delivery (optimisation of program structure) and supervision needs. Adherence to exercise interventions in people with dementia in the aged-care setting varies from 25.5 to 84% with high variability between studies [23]. Our previous study showed 80–86% adherence to the Seniors Exercise Park exercise program [17, 19]; however, we anticipate some potential barriers for participation (e.g. mental wellbeing, anxiety, depression, decreased mobility) as reported for people living with dementia [23]. We are aiming for an estimated participation rate of ≥ 70% of the prescribed number of exercise sessions [24] during the 12-week structured exercise program, and ≥ 60% adherence for the overall 6-month exercise program. Retention of 85% of the sample is expected (with estimate 15% drop out) at 6 months.

##### Safety

Safety will be assessed based on the following: any falls that occur during the exercise delivery sessions, any adverse events (joint/muscle pain during the exercise, or as a result of the exercise sessions), serious adverse events (any report of difficulty breathing that does not settle quickly with rest, new or unrelenting chest pain, or acute changes in the level of consciousness during the session where a medical emergency procedure is required). Any incidents and or events will be recorded at each session.

##### Physical activity program optimization/adaptations to the program structure

The Seniors Exercise Park program is a structured 12-week exercise program with incremental increase in the number and/or type of exercises and exercise duration throughout the 12 weeks [24]. The exercise program has been delivered to over 80 older people without cognitive impairment who are living in the community. Any modifications to the delivery of the overall exercise program and/or individual exercise sessions (e.g. exercise length, session duration) due to safety concerns, and other reasons (e.g. mental or physical fatigue) will be documented. During the maintenance phase (exercise after completion of the formal research supervised component, from 12 to 24 weeks post baseline), the exercise behaviors of each participant (activity and duration) will be monitored and recorded by the exercise instructor and by a supervisor from the facility (e.g. facility staff will encourage and supervise ongoing participation and use of the exercise park equipment). Field notes (post-session reflection notes) will be completed by the research staff noting down any variations of the program, participant’s behavior and any other relevant aspects identified by the staff.

##### Supervision needs

Our previous and current research suggests that one supervisor for 6–10 participants who are functionally independent (no walking aid, or with limited usage of walking aid) is safe. We anticipate that some people will have mobility limitations as well as cognitive impairment, and therefore will aim initially for one qualified instructor (exercise physiologist/physiotherapist) and another supervisor (from the aged-care staff) for up to 3–4 participants. As we expect participants to improve throughout the exercise program, the supervision ratio will be reassessed every two weeks and be adjusted accordingly. The number of participants at each session and number of supervisors will be recorded and monitored throughout the program, and will be used in analysis and reporting.

#### Secondary outcomes

A comprehensive suite of physical function (strength, balance, functional mobility), psychosocial (quality of life, depression, loneliness), cognitive, and falls risk and falls history will be assessed. Physical activity participation, engagement, social interaction, mood and enjoyment data will also be collected.

#### Physical function measures

Physiological measures of strength, balance and functional mobility will be assessed using the following validated tests:

(i) Functional lower limb muscle strength using the five times sit to stand test [25]. Participants will be asked to sit on a chair (43 cm high) and stand up (with arms crossed over their chest) five times. The time taken to complete the sit to stand will be measured (seconds).
(ii) Exercise tolerance and functional mobility using the two-minute walk test [26]. Participants will walk in a marked area for 2 min at their comfortable pace. The distance covered during the 2 min will be recorded. The length of walkway will be around 10–15 m, based on available length of the facility corridor.
(iii) Dynamic balance using the step test [27]. Participants will be asked to place one foot onto a 7.5cm-high step and then back down to the floor repeatedly as fast as possible for 15 s. The number of steps completed in the 15s period for each leg will be recorded. The step test is a test of dynamic standing balance, only takes approximately 1 min to administer, has been shown to have good retest reliability (ICC = .868) in people living with dementia [28], and demonstrated significant improvement in another Seniors Exercise park study (ENJOY translation study) in older people without cognitive impairment [19].
(iv) Walking speed using the 4 meters walk test [29]. Participants will be asked to walk 4 m at their usual walking pace. Gait speed will be defined by distance (in meters) divided by time (in seconds). Participants will start walking 2 m before the 4m walkway, and finish walking 2 m after the 4m walkway.

#### Psychosocial, cognitive and quality of life health outcomes

A set of valid, reliable and widely used instruments (specifically for older people and those living with dementia) for cognitive screening, quality of life, socialisation, daily activity engagement and depression will be used.

(i) *The Montreal Cognitive Assessment (MoCA)* is a brief 30-question cognitive screening test. It assesses several cognitive domains: attention and concentration, executive function, memory, language, visuoconstructional skills, conceptual thinking, calculations and orientation [30].
(ii) *Health-related quality of life* will be assessed using two instruments: the Quality of Life in Alzheimer's disease scale (QoL-AD) and the EQ-5D-5L. The QoL-AD will be administered via interview with the person and is comprised of 13 items (physical health, energy, mood, living situation, memory, family, marriage, friends, self as a whole, ability to do chores, ability to do things for fun, money and life as a whole) [31]. The EQ-5D-5L is a generic instrument to assess health related quality of life that comprises five dimensions (mobility, self-care, usual activities, pain/discomfort and anxiety/depression) as well as overall utility score. The EQ-5D-5L allows comparison across multiple populations [32], and is the most widely published and used health utility scale for which there are multiple utility conversions available for populations internationally.
(iii) *Fear of falls* will be assessed using the Iconographical Falls Efficacy Scale [33]. The IFES is a valid and reliable 10 items scale that assesses fear of falling in older people with cognitive decline.
(iv) *Loneliness* will be assessed using the UCLA 3-Item Loneliness Scale. The UCLA Loneliness Scale includes three dimensions of loneliness: relational connectedness, social connectedness and self-perceived isolation [34, 35].
(v) *Depression* will be assessed using the short version Geriatric Depression Scale (GDS-15) [36]. The GDS-15 is a 15-item instrument where a score of 0 to 5 is considered normal and a score greater than 5 suggests depressive symptoms.
(vi) *Social isolation and social support* will be assessed using the short version 6 items Lubben Social Network Scale (LSNS-6) [37]. The score ranges between 0 and 30 where higher scores indicate more social engagement
(vii) *Engagement with daily activity* will be assessed by aged-care staff using the Pool Activity Level (PAL). The PAL is a widely used, validated, measure of engagement with activity for older people living with dementia [38]. The researchers/aged-care staff will rate residents’ ability to plan and perform during nine common daily activities—bathing/washing, dressing, eating, contact with others, group work skills, communication skills, practical activities, use of objects, reading/looking at a newspaper/magazine. Each activity is scored on a 4-point scale: 1 = ‘planned—needing little or no help with everyday activities’; 2 = ‘exploratory—able to carry out familiar activities in familiar surroundings’; 3 = ‘sensory—limited ability to perform an activity’; 4 = ‘reflex’—unable to perform basic everyday tasks without assistance’. The caregiver ticks the one that most accurately describes the individual’s performance of that activity over the preceding two weeks.

#### Falls risk assessment

(i) *The Falls Risk Assessment Tool (FRAT)* will be used to assess fall risk [39]. The FRAT is a 4-item falls-risk screening tool for sub-acute and residential care: with risk factors scored to reflect graded risk of low, moderate and severe risk.

#### Perceptions and feedback of the physical activity program –from aged-care staff and from participants

##### During the physical activity program delivery

Mood and enjoyment will be recorded *from the participants* and will include the following:

*Mood—*This will be assessed at the beginning of each session and will include a 5-point Likert scale using a visual smiley face card as follows: 0—sad, depressed, down; 2—in the middle (not happy, not sad) and 4—happy (high, awesome, great).

*Enjoyment*—At the end of the exercise session participants will be asked if they enjoyed the program, and to rate their level of enjoyment on a 5-point Likert scale using a visual smiley face card as follows: 0—not at all, 2—in the middle (neutral), 4—enjoyed it very much (had fun).

During the physical activity program motivation to participate, engagement and wandering behavior will be recorded *by the staff* (research instructor, facility staff) as follows:

*Motivation to participate*—participants’ motivation will be assessed by the exercise supervisor at each exercise session based on their observation and interpretation of participants’ expression, verbal prompt and body language [40]. A 5-point Likert scale will be used as follows: 0—no motivation (present without participating), 1—low motivation (needs to be convinced to participate), 2—moderate motivation (attends the activity without being positive or negative), 3—high motivation (participates with positivity), and 4-very high motivation (participates very positively) [40].

##### Group observational measurement of engagement (GOME)

The GOME will be used to evaluate engagement in the physical and social activity and includes engagement on an individual and group levels [41]. On an individual level using a Likert scale, the engagement assessment includes one variable pertaining to participation in the group (attendance duration), three variables that assess engagement levels (engagement—amount of time participants are attentive to the group activity; active participation, and attitude toward the activity) and one variable that assesses sleep or sleep-like symptoms during the group activities for each participating individual.

On a group level, engagement will include record of number of participants in the group, positive (smiling, positive comments to others) and negative (angry comments toward group members) interactions using a 6-point Likert scale.

##### Wandering behavior

In circumstances where participants suddenly stop exercising without any particular reason (e.g., unrelated to fatigue, muscle soreness) and start wandering (walking off) around the park area, a staff member will ensure their safety and assist them to return to the park or to be seated in the resting area. Participants who walk off will be intermittently encouraged to re-join the exercise group. The staff will also document the behavior (description, duration and time occurred).

##### General motivation and engagement behavior at selected time points throughout the program

Motivation to go to the activity will be assessed at 5 time points: at the beginning of the structured physical activity program (week 1), halfway through (week 6) and at the end (week 12), halfway through the maintenance phase (week 18), and at the completion of the maintenance phase (week 24).

Motivation will be assessed using a 5-point Likert scale [0, very negative (never wants to or usually does not want to go to sessions, despite motivating attempts); 1, negative (needs to be motivated to go to sessions, which they usually do); 2, neither positive nor negative (goes to sessions without being motivated); 3, positive; 4, very positive] [40].

##### Overall feedback

At the completion of the 12-week exercise intervention, participants will be asked about their overall experience and to provide feedback about the exercise program. Feedback from the aged-care staff (staff who are involved in the project delivery) will also be collected at the completion of each exercise group using a survey (a questionnaire incorporating open ended and rating scale questions).

### Exercise Park intervention

#### The Seniors Exercise Park equipment

**Fig. 2:**
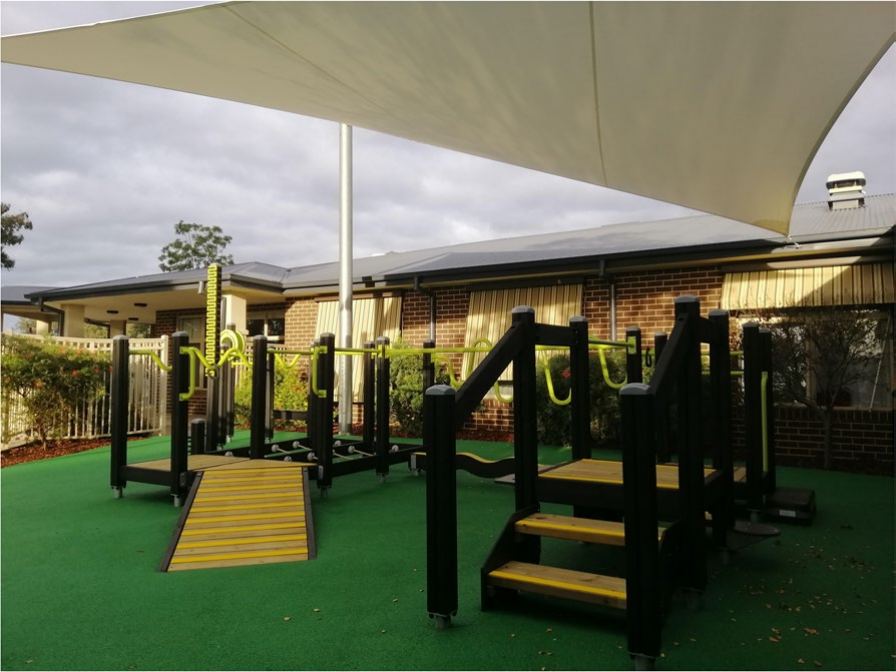
The Seniors Exercise Park

The exercise park equipment is outdoor playground equipment specifically designed for older people to improve strength, balance, joint movements, and mobility and function (Fig. 2). It comprises multiple equipment stations that target specific function or movement (upper and lower limb) such as shoulder range of movement, static and dynamic balance, and functional movement such as walking up/down stairs and sit to stand. The ground surfaces are non-slippery rubber (softfall) suitable for any playground equipment (Fig. 2). The area is also covered by waterproof sail shade cover.

The equipment is located outdoors and safe to use by any age group. Our previous studies found the exercise park to be safe for older people (aged 60 years and over, including with increased risk of falls) with no adverse events [17, 19]. The exercise park equipment was installed in 2019 at Liscombe House, Leith Park, and is owned and managed by the aged-care facility.

#### The Seniors Exercise Park program

##### 12-week structured supervised exercise program

Participants will undergo a 12-week supervised exercise intervention program twice a week using the Seniors Exercise Park. Participants will perform exercises that focus on strength, balance, coordination, mobility and flexibility as detailed in our previous work [15, 24]. Examples of the exercises can be found here https://youtu.be/PaYuCMtnlYk. The exercise park sessions will be provided two times a week (each class approximately 1 to 1.5 h duration) and will be supervised by a qualified accredited exercise physiologist or physiotherapist with the assistance of another supervisor (aged-care staff; physiotherapist/diversional therapist). Each session will consist of 5–7 min warm-up exercises, followed by 45–75 min on the equipment stations, and will conclude with 5–7 min of cool down exercises. The exercise classes will include 3–4 participants and will be circuit-based. To maximise social interaction and enjoyment, morning/afternoon tea will be organised following the exercise sessions.

##### Exercise intensity

The exercise progression will follow the guidelines of the Australian Position Statement on exercise for falls prevention [42]. The initial level of the exercise difficulty will be tailored to the capabilities of the participant with the primary consideration of safety. Adjustment of the exercises (i.e. increase in intensity and difficulty) will be made based on the individual participant progression. Additional exercises will be gradually introduced to the participants every 1–2 weeks. The supervising staff will determine timing of exercise progression based on clinical judgement including consideration of levels of apparent fatigue and exercise difficulty. Participants will also be asked how difficult the exercise was for them and how they generally feel when they perform it, which will further assist the staff to adjust the exercise to the individual.

##### Individual and group exercise progression

Each exercise station will include two exercises and will be performed twice by each participant. Two participants will be allocated in each station, such that each participant will perform one at a time and will swap over. Participants will be given a resting period of 30–60 s (or longer if needed), which will be adjusted according to program progression. The duration of each exercise will also increase based on program progression. Participants will be allowed to have as many breaks as necessary to keep them performing the exercises with good technique and proper form. Examples of exercise duration, rest and progression are given in Table 2. The general exercise program structure will follow our previous published protocol [15, 24]; however, given this is a feasibility study with a different clinical cohort, we anticipate modifications in the program and will be recording and reporting any variations.

**Table 2:** Duration of exercise time, rest time and progression

Week number	Set time	Rest time
1 to 2	60 s	30–60 s to change over and rest
3 to 5	60 s	≤ 30 s change over and rest
6 to 9	75 s	≤ 30 s change over and rest
10 to 12	90 s	≤ 30 s change over and rest

#### Exercise uptake and physical activity maintenance

##### Maintenance phase: 12–24 weeks post-baseline

After completion of the 12-week program, it is expected that participants will be familiar with the equipment and the exercises and therefore able to exercise more independently. Pre-scheduled sessions will be available for participants to access and use the Seniors Exercise Park under supervision (aged-care/diversional therapist staff from the facility). To facilitate independence and empowerment, participants will be encouraged to exercise independently (supervised but unstructured sessions). Small exercise cards with graphic images of the required exercises will be placed in each exercise station to aid participants to progress through the stations.

### Control group

Participants from the control group will be given the opportunity to participate in organised recreation and leisure-based group activities, such as art or music, that are run by the Diversional Therapist staff at the aged-care facility. The exercise intervention provided is in addition to what is already provided and is not replacing any other physical activities, therefore control participants will not be withheld from receiving physical activity programs or any related one-on-one physiotherapist sessions.

### Safety considerations and adverse events

#### Wandering behavior

Wandering or ‘walking off’ behavior is common amongst people living with dementia and is defined as the inability of older adults with dementia to find their way while pursuing a need or goal [43]. Walking off may occur during the delivery of the exercise program. A staff member from the facility (or the exercise instructor) will be taking the participants to and from the exercise park area at the beginning and at the completion of the session. Importantly, the Seniors Exercise Park is fenced with a locked gate. If deemed necessary, during the exercise session delivery, the gate will be shut to prevent participants from leaving the area. The area has a seated bench and resting chairs where participants can sit down and rest. Participants who walk off will be intermittently encouraged to re-join the exercise group.

#### Weather conditions

In extreme weather conditions (e.g. heavy rain, extreme hot days (above 35 °C)) if deemed by the exercise instructor as unsafe to exercise, sessions will be cancelled. In summer in Melbourne, classes will be conducted in the morning and late afternoon (to avoid the high temperatures during the middle of the day), and shade-cloth cover and/or other sun-smart behaviors will be facilitated. Sunscreen and water will be available from the exercise instructor at supervised sessions. In circumstances where sessions are cancelled, re-schedule of sessions will be attempted (if weather and staff/participant availability permit); or make up sessions will be organised towards the end of the program.

#### Adverse events

The exercise program is designed to be moderate intensity, where exercise difficulty is tailored to each participant based on their abilities. Given that we aim to test the feasibility and safety of the exercise program in residents living with dementia, we expect that adjustments will occur based on how the participants respond at each session.

##### Muscle soreness/joint pain/discomfort

Following the exercise sessions, some muscle soreness is anticipated and is a typical response of the body to exercise in people who have done little, if any recent exercise, or people who are performing an unfamiliar type of exercise. Instances of severe muscle soreness (muscles soreness that does not settle within a few days) reported by the participant will be recorded.

##### Joint pain

It is anticipated that some participants might have pre-existing musculoskeletal comorbidities such as joint arthritis or join pain (for example at the knee or hip). In instances where participants report increased joint pain, the exercise will be reviewed and modified. Any severe pain (more than 7 on a 0–10 pain scale) that is persistent over 2–3 consecutive sessions, will be noted and the exercise program will be modified for that participant.

##### Falls

Any falls occurring during the exercise sessions will be recorded. A fall is defined as an event when the participant ‘inadvertently comes to rest on the ground, floor or other lower level’ (WHO Global Report on Falls Prevention in Older Age [44]).

##### Cardiorespiratory adverse reaction

Any report of difficulty breathing that does not settle quickly with rest, new or unrelenting chest pain, or acute changes in the level of consciousness during the session, may precede a serious medical emergency. A potentially serious event will be defined if the participant reported difficulty breathing but symptoms settled quickly with rest and their clinical signs (respiration rate, heart rate, oxygen saturation) remain normal. A serious adverse event will be defined if symptoms have not settled and medical emergency care was required.

##### Data storage, management and dissemination

All participants will be assigned a code/ID. The information will only be accessible to the research team. All hard copies of the assessments and questionnaires will include participants code/ID and not their personal information and will be kept on a locked cabinet in a locked research team office. Information stored on computer files will be password protected. Field notes, such as adherence sheets and other information that will be recorded during the exercise session delivery will include minimal personal identification (e.g. name of participant) but without any sensitive information or any identification code linking to the participants. These field notes will need to be used on a regular basis and will be kept with the researchers or in a secure cabinet at the aged-care facility.

At the completion of the project, the publications or research reports will be available to participants or their consenting person on request. Copies of the publications will be given to the aged-care staff who will also be able to share it with the residents. A one page plain language summary of the project results will be provided to all study participants and /or their family.

##### Cleaning and hygiene practice—a standard precaution for infection control (including COVID-19)

All staff will practice good hand hygiene when arriving on site including washing hands and using hand sanitiser. Hand sanitisers will also be available for both staff and participants to use at the beginning and completion of each exercise session, or during exercise sessions if required. The equipment (handles) will be regularly cleaned using detergent solution/disinfectant spray or wipes.

### Statistical methods

#### Sample size estimation and justification

This is a feasibility study with the aim of designing, delivering and evaluating a sustainable physical activity program for people living with dementia in residential aged-care. Leith Park residential aged-care facility has approximately 80 residents, with 37 residents living with dementia (diagnosed as mild to severe dementia by a geriatrician). For the pilot study, we aim to recruit 12 participants for each group. We anticipate 3–4 exercise groups, with a total of 24 participants overall (20 participants completing the study allowing for ~ 15% drop out rate). Our previous trial had 11% drop out from the exercise intervention group [17]. The targeted sample is feasible to be recruited and then followed up for 6 months to allow completion of the trial. The targeted number is also sufficient to provide preliminary results about the feasibility and safety of the exercise program to then optimise it for people living with dementia.

It should be noted that the study is not powered for significant changes in the secondary measures, but that the data from this study will inform sample size calculation for a future large multi-site cluster randomised trial.

### Statistical analysis

Data for the feasibility and safety components of the study will be analysed as follows (mean, standard deviation and proportion): proportion of participants approached to participate who did commence the program, percentage of participants who complete the intervention, overall percentage of sessions attended, number of participants who dropout, any falls that occur during the exercise sessions, any muscle/joint pain during or after the exercise sessions, and serious adverse events requiring medical attention. Modifications of the exercise program will be reported and a recommended refined structure will be presented at the end of the program.

An acceptable level for feasibility of the program will be based on the following: ≥ 70% adherence of the prescribed number of exercise sessions [24] at the 12-week structured exercise program completion, and a ≥ 60% adherence for the overall 6-month exercise program, with retention of 85% of the sample (estimate 15% drop out).

To determine statistical trends of effectiveness, analyses of the selected outcomes such as cognitive, physical function, quality of life and social measures will be performed to assess the changes within and between groups over time (pre/post). Repeated measures ANOVA with factors of intervention (Seniors Exercise Park program, control) and time (pre-post intervention and follow-up) will be used. A post hoc analysis will be undertaken using the group differences and variability within groups on the secondary measures to determine the required sample size for a future adequately powered study using these outcomes.

## Discussion

Although the underlying neuropathology of dementia is not curable, lifestyle factors such as social and physical activities can influence the mental health, quality of life and rate of cognitive decline in people living with dementia, even after diagnosis. Given the growing numbers of people living with dementia, there is a need to create changes to communities and aged-care facilities to make them more dementia friendly and to support people living with dementia to continue to live well. In this pilot study, we take an innovative approach to engage people living with dementia in physical and social activity program. The Seniors Exercise Park equipment, installed and launched in June 2019 at Leith Park in Melbourne, is the first of its kind in a residential aged-care setting. The exercise program provides a fun but physically challenging environment to support exercise in residential aged-care settings, addressing key aspects of physical health for older people, including strength, balance, mobility and function. While the exercise park was designed to actively promote community well-being through the provision of a unique exercise and social support program, this has never been tested and applied to older people living with dementia. This project will create an active outdoor environment that is not just age friendly but also dementia friendly.

It is important to acknowledge that this is a pilot study and the results might be limited to provide emerging evidence around the suitability of the program for people living in dementia in aged-care. However, the outcomes will generate new evidence around the design of a suitable physical environment and associated exercise program to improve quality of care and quality of life for people living with dementia in residential care in Australia. If successful, the intervention represents a safe, accessible, affordable and highly translatable program, and results could inform future design of the built environment for residential aged-care facilities. In addition, the secondary outcome measures to be assessed in the study will provide preliminary results around the potential effect of the Seniors Exercise Park program on physical and cognitive function to inform a future fully powered study design to assess effectiveness. The Seniors Exercise Park is also scalable as it is available in many countries in the world including the USA and Canada, enabling wide reach to be replicated in other aged-care settings around the world. The real-world setting of this study will enable practical information and knowledge to be generated to enable potential implementation of the program and equipment in existing care facilities worldwide.

## Data Availability

Not applicable.

## Abbreviations

ENJOY: Exercise interveNtion outdoor proJect in the cOmmunitY
QoL-AD: Quality of Life in Alzheimer's disease scale
EQ-5D-5L: The 5-level EQ-5D
sMMSE: Standardised Mini-Mental State Examination
UCLA: University of California at Los Angeles
MoCA: Montreal Cognitive Assessment
GDS: Geriatric Depression Scale
PAL: Pool Activity Level
IFES: Iconographical Falls Efficacy Scale
FRAT: The Falls Risk Assessment Tool
GOME: Group Observational Measurement of Engagement
